# Safety and efficacy of rapid withdrawal of anti-seizure medication during long-term video-EEG monitoring

**DOI:** 10.3389/fneur.2023.1196078

**Published:** 2023-07-11

**Authors:** Jiao Liu, Deng Chen, Yingchun Xu, Yu Zhang, Ling Liu

**Affiliations:** Department of Neurology, West China Hospital, Sichuan University, Chengdu, Sichuan, China

**Keywords:** epilepsy, anti-seizure medication, rapid withdrawal, pre-surgical evaluation, long-term video-EEG monitoring

## Abstract

**Objective:**

Anti-seizure medications (ASMs) are often withdrawn during long-term video-EEG monitoring (LTM) to allow pre-surgical evaluation. Herein, we evaluated the safety and efficacy of ultra-rapid withdrawal (URW) and rapid withdrawal (RW) of ASMs in an epilepsy monitoring unit (EMU).

**Methods:**

This retrospective study examined all consecutive patients admitted to our EMU between May 2021 and October 2022. Patients were classified into the URW and RW groups according to the way ASMs were withdrawn. We compared the efficacy and safety of the procedures used in the groups in terms of duration of LTM, latency to the first seizure, and incidence of focal to bilateral tonic–clonic seizures (FBTCS), seizure clusters (SC), and status epilepticus (SE).

**Results:**

Overall, 110 patients (38 women) were included. The mean age of patients at the time of LTM was 29 years. All medications were stopped on admission for monitoring in the URW group (*n* = 75), while in the RW group (*n* = 35) ASMs were withdrawn within 1 day. In both groups, the duration of LTM was approximately 3 days: URW group (2.9 ± 0.5 days) and RW group (3.1 ± 0.8 days). The latency to the first seizure was significantly different between the two groups; however, there were no differences between the two groups in terms of the distribution of FBTCS, SC, or SE, number of seizures, and the requirement for intravenous rescue medication was low.

**Conclusion:**

The rapid withdrawal of ASMs to provoke seizures during monitoring for pre-surgical evaluation following the URW protocol was as effective and safe as with RW. Ultra-rapid ASM withdrawal has the benefits of reducing LTM duration and shortening the time to first seizure compared to rapid medication tapering.

## Introduction

Long-term video-electroencephalography monitoring (LTM) in the epilepsy monitoring unit (EMU) can be used to localize the irritative region and site of seizure onset during surgical evaluation (1). Seizure events are unpredictable and infrequent and failure to capture them and accurately locate the epileptogenic zone may result in repeated admissions, increased cost of hospitalization and anxiety in patients (2). Withdrawal of anti-seizure medications (ASMs) and sleep deprivation are common approaches to provoke seizures to facilitate LTM within a limited time (3). However, ASM withdrawal is associated with an increased risk of prolonged seizures, focal to bilateral tonic–clonic seizures (FBTCS), seizure clusters (SC), and status epilepticus (SE) (4–6).

No clear consensus exists on the optimal protocol for ASM withdrawal during LTM for pre-surgical evaluation, and the description of the specific approaches for medication taper is frequently omitted in several studies. The rate of medication withdrawal is related to many factors, including the number and type of ASMs being tapered, and habitual seizure rate. The EMU admission protocols include various ASM withdrawal methods that are gradually initiated following admission. The average length of EMU stay to achieve the goal of admission is generally less than a week, despite the speed of the withdrawal (2, 4, 7).

Previous studies have revealed the reduction of the initial ASM dose by 30 and 16% daily as rapid and slow tapering, respectively (8, 9). Moreover, rapid ASM withdrawal may increase the risk of complications, such as falls, SC, and SE. Considering the lack of literature on this topic, further studies are required to clarify the conditions under which ASM cessation for seizure evaluation is likely to be both safe and effective. This study presents a retrospective evaluation of the safety and efficiency of rapid ASM withdrawal.

## Patients and methods

### Subjects

All adult patients with epilepsy admitted to the EMU at the West China Hospital of Sichuan University for pre-surgical evaluation were recruited from May 2021 to October 2022. Patients under the legal age, mentally disabled or significant nonneurologic comorbidities were excluded from the study. All participants provided written informed consent, and agreed to follow the assigned protocol for acute withdrawal of ASMs. This study was approved by the Ethical Committee of the West China Hospital of Sichuan University.

### Definitions

SC was defined as the incidence of three or more seizures within 4 h or 24 h, with a return to baseline between events (9, 10). SE was defined as ≥5 min of tonic–clonic convulsions, ≥10 min of focal seizures with impaired consciousness, ≥10 min of ictal electrographic activity, or a series of seizures with no return to the neurological baseline between events (11, 12). Patients with both SE and SC were assigned to the SE group and the group with more severe complications. The number of SC events, and number of seizures in each SC were obtained on review of the EEG recordings by an epileptologist. An LTM was defined as successful when the presurgical team found no need to repeat it to arrive at a conclusion, including proceeding to surgery or denying surgery; A registration of at least three habitual seizures would be typically required. The number of ASMs prescribed to patients before LTM includes all medications prescribed from the onset of the disease until now, while the number of ASMs on admission refers to the current medication.

### Video-EEG monitoring

Presurgical evaluation usually includes up to 3 days of continuous LTM in the EMU. The limitation of 3 days at our unit was due to regulations aiming to ensure optimal use of the EMU capacity. Exceptionally, when there was a vacancy in the EMU schedule, patients without enough seizures were able to continue after having completed 3 days. LTM was continued or ceased as per the decision of the epileptologist until an adequate number of seizures (usually more than three) with a good visual EEG quality were recorded.

### ASM withdrawal

Patients were assigned to the ultra-rapid withdrawal (URW) group if all medications were stopped on the first day of LTM, and to the rapid withdrawal (RW) group if half of the ASM were tapered on day 1 and stopped on day 2 over the course of hospitalization. All patients included in the study tapered their ASMs and were assigned to the URW or RW groups. The rapid withdrawal of ASMs was determined by the physician in charge.

All patients were prepared with an intravenous line prior to initiating the LTM recording to facilitate the immediate administration of benzodiazepine (i.v. diazepam or midazolam) or phenobarbital in the event of SC or SE. All ASMs were resumed on the day of completion of monitoring.

### Statistical analysis

Continuous data are presented as the mean ± standard deviation, and were analyzed using the Student’s t-test. Categorical data are presented as counts (percentages) and were analyzed using the chi-square test or Fisher’s exact test. A two-tailed *p* value <0.05 was considered statistically significant. All analyzes were performed using SPSS 21.0.

## Results

### Patient characteristics

A total of 337 patients, known or suspected cases of epilepsy, were admitted to the EMU in our center for LTM between May 2021 and October 2022. After excluding juvenile patients, psychogenic non-epileptic seizures patients and patients who refused the rapid withdrawal of ASMs, only 110 participants met study criteria and were included in the analysis. The study cohort included 63 (65%) men, and the mean age and disease duration of the cohort were 29 y (Standard deviation [SD] = 9 y; range: 18–59 years) and 12 y (SD = 9 y; range: 0.5–45 years), respectively. In total, 75 and 35 patients were included in the URW and RW groups, respectively.

**Figure 1:**
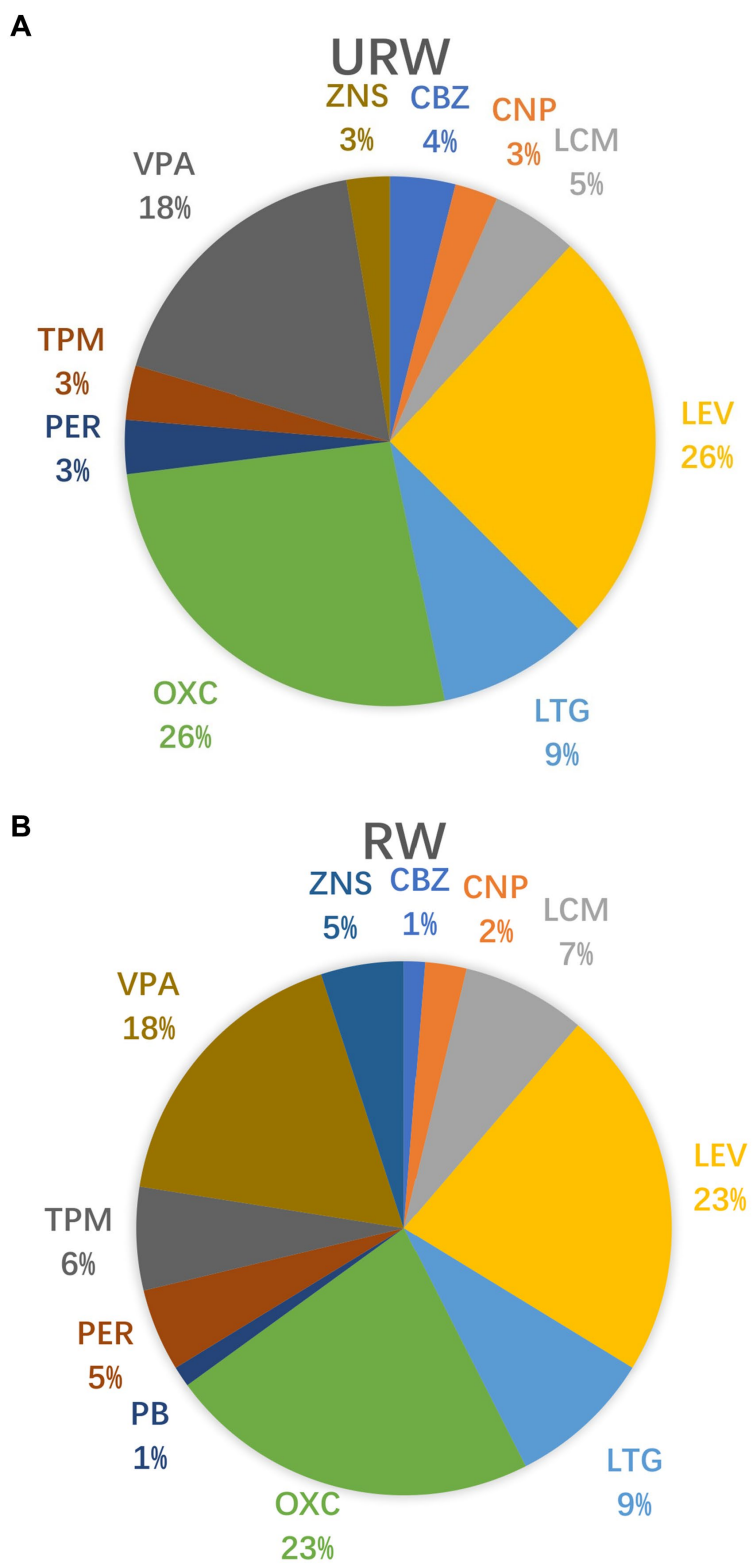
ASMs taken on admission in the URW and RW groups. **(A)** The type of ASMs in the URW group. **(B)** The type of ASMs in the RW group. CBZ (carbamazepine), CNP (clonazepam), LCM(Lacosamide), LEV (levetiracetam), LTG (lamotrigine), OXC (oxcarbazepine), PB (Phenobarbital), PER(Perampanel), TPM (topiramate), VPA (valproic acid), ZNS (zonisamide).

The demographic characteristics of the patients are listed in Table 1. The age at seizure onset in patients in the URW group was significantly higher than that in the RW group, while the duration of epilepsy tended to be shorter (*p* < 0.05). No statistical differences were reported in the sex, age at LTM, monthly seizure frequency, history of FBTCS, history of SC/SE, magnetic resonance imaging (MRI) brain findings, or number of ASMs prescribed before LTM and on admission between the URW and RW groups. The most common findings on brain MRI and positron emission tomography MRI (PET-MRI) in the URW group were mesial temporal sclerosis (*n* = 27), followed by focal cortical dysplasia (*n* = 9), cavernoma (*n* = 7), and normal brain MRI results (*n* = 7). The most common finding in the RW group was mesial temporal sclerosis (*n* = 13), followed by normal brain MRI results (*n* = 4), and encephalomalacia (*n* = 4). The median number of ASM on admission was 2.08 (range: 1–4). As shown in Figure 1, the five most commonly used ASMs by patients in both groups were levetiracetam, oxcarbazepine, valproic acid, lamotrigine, and lacosamide.

**Table 1:** Demographic and clinical characteristics of patients.

	Ultra-rapid Withdrawal (*n* = 75)	Rapid Withdrawal (*n* = 35)	*p* value
Men (%)	47 (62.7%)	25 (71.4%)	0.36
Age at LTM, years	29.15 ± 9.67	27.26 ± 8.27	0.32
Age at seizure onset, years	18.34 ± 11.14	12.51 ± 9.21	**0.008**
Duration of epilepsy, years	10.77 ± 8.11	14.74 ± 9.36	**0.02**
Monthly seizure frequency	6.86 ± 14.41	5.26 ± 6.72	0.54
MRI brain findings			
Normal	7 (9.3%)	4 (11.4%)	0.73
Mesial temporal sclerosis	27 (36%)	13 (37.1%)	0.91
Focal cortical dysplasia	9 (12%)	3 (8.6%)	0.59
Encephalomalacia	6 (8%)	4 (11.4%)	0.56
Cavernoma	7 (9.3%)	1 (2.9%)	0.22
Traumatic brain injury	2 (2.7%)	1 (2.9%)	0.95
Tumor	2 (2.7%)	0 (0%)	NA
Other	15 (20%)	9 (25.7%)	0.50
Number of ASMs			
1	20 (26.7%)	4 (11.4%)	0.07
2	38 (50.7%)	19 (54.3%)	0.72
3	15 (20.0%)	10 (28.6%)	0.32
4	2 (2.7%)	2 (5.7%)	0.43

Bold values indicate statistical significance of *p* < 0.05.

LTM, long-term video-EEG monitoring; FBTCS, focal to bilateral tonic–clonic seizures; SC, seizure clusters; SE, status epilepticus; ASMs, anti-seizure medications; MRI, magnetic resonance imaging.

### Efficacy of rapid medication withdrawal

All medications were stopped on admission for LTM in 75 patients (68.2%), and the ASM of the RW group was withdrawn within 3 day in 35 patients (31.8%). Twenty patients in the URW group and five patients in the RW group did not have any seizures during LTM (Table 2). The mean duration of LTM was 2.9 days (SD = 0.5 days; range: 2–5 days) in the URW group, and 3.1 days (SD = 0.8 days; range: 2–6 days) in the RW group, with no significant difference (*p* = 0.14). Due to vacancies, 12 patients with few seizures during the first 3 days were able to extend their stay during LTM. A statistically significant difference was reported in the time to first seizure between URW (19.7 ± 18.5 h) and RW (30.3 ± 14.7 h, *p* = 0.008) groups. However, the percentage of patients with successful localization of ictal onsets (*p* = 0.78), number of seizures (*p* = 0.20), daily seizure frequency (*p* = 0.80), daily FBTCS frequency (*p* = 0.26), focal-aware seizures (FAS, *p* = 0.57) and focal impaired awareness seizures (FIAS, *p* = 0.57) did not differ significantly between the two groups. Overall, 52 patients (47.3%) underwent epilepsy surgery, including 33 patients in the URW group and 19 patients in the RW group, while 3 patients underwent stereotactic electroencephalography (SEEG).

**Table 2:** Results of seizures occurring during the LTM.

Results	Ultra-rapid withdrawal (*n* = 75)	Rapid withdrawal (*n* = 35)	*p* value
Duration of LTM, days	2.9 ± 0.5	3.1 ± 0.8	0.14
Latency to the first seizure, h	19.7 ±18.5	30.3 ± 14.7	**0.008**
Number of seizures	9.35 ± 20.31	4.47 ± 3.20	0.20
% of patients with ictal onset EEG localization	45 (60.0%)	22 (62.9%)	0.78
Daily seizure frequency	2.49±6.12	1.31±1.25	0.26
Daily FBTCS frequency	0.32±0.66	0.29±0.42	0.80
No seizures during monitoring	20 (26.7%)	5 (14.3%)	0.15
Seizure type			0.57
FIAS	43 (57.3%)	25 (71.4%)	
FAS	12 (16.0%)	5 (14.3%)	
FBTCS	23 (30.7%)	14 (40.0%)	0.34
4-h SC	18 (24.0%)	5 (14.3%)	0.24
24-h SC	31 (41.3%)	14 (40.0%)	0.90
SE	1 (1.3%)	1 (2.9%)	0.58
Rescue medication use^a^	6 (8.0%)	5 (14.3%)	0.31

Bold values indicate statistical significance of *p* < 0.05.

a Rescue medication use included intravenous diazepam, midazolam, or phenobarbital.

LTM, long-term video-EEG monitoring; FAS, focal-aware seizures; FIAS: focal impaired awareness seizures; FBTCS, focal to bilateral tonic–clonic seizures; SC, seizure clusters; SE, status epilepticus.

### Safety of rapid medication withdrawal

**Figure 2:**
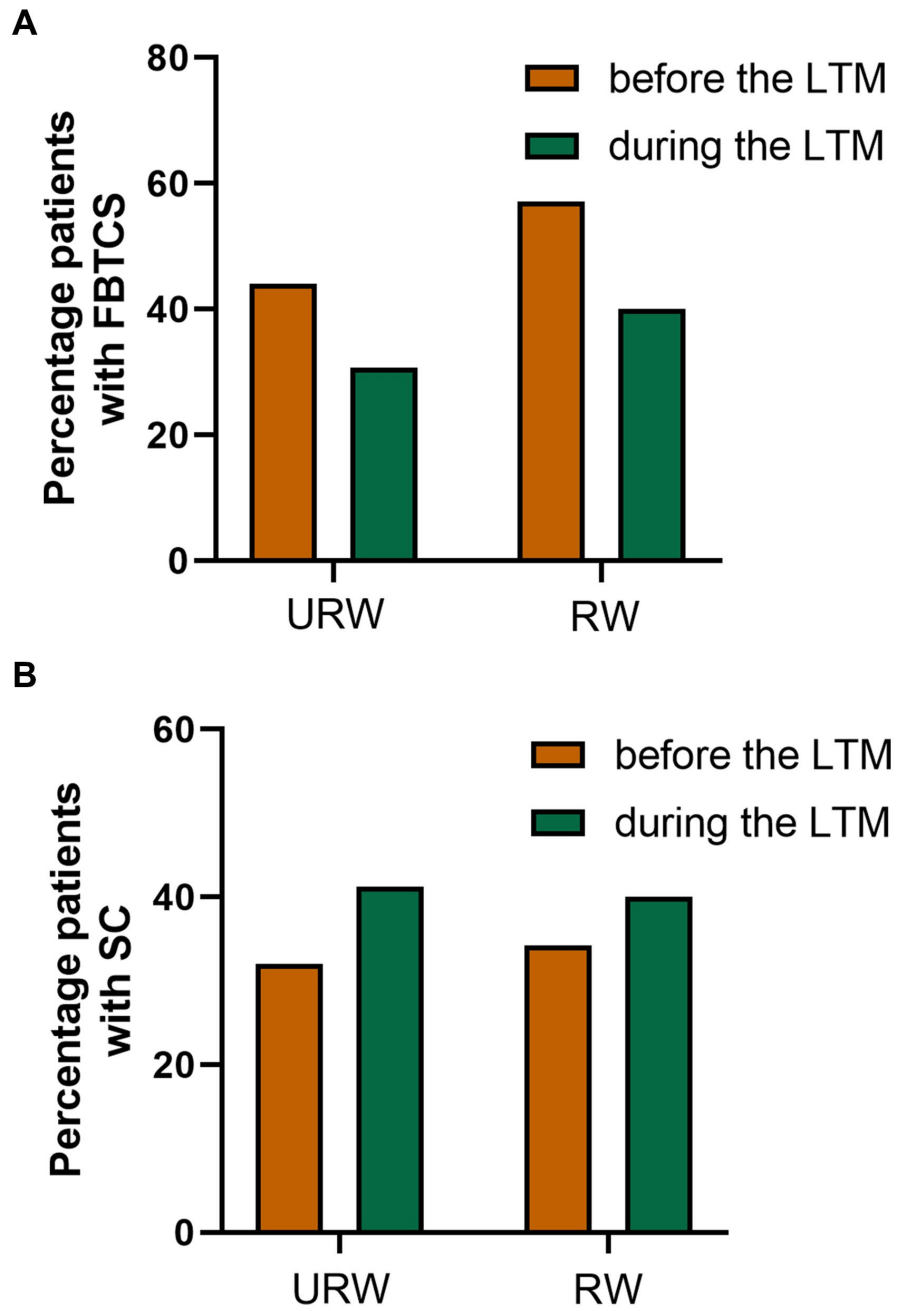
Results of seizures occurring before and during the LTM. **(A)** Percentage patients with FBTCS before and during the LTM. **(B)** Percentage patients with SC before and during the LTM.

Among all patients included in the study, 45 (40.9%) experienced at least one SC and two (1.8%) had at least one episode of SE, while 63 (57.3%) experienced no seizure complications during LTM. Further, 23 (30.7%) and 14 (40.0%) patients in the URW and RW groups presented FBTCS, respectively (Table 2). In the URW group, 18 patients (24.0%) presented with 4-h SCs, and 31 patients (41.3%) presented with 24-h SCs in the LTM. In the RW group, 5 patients (14.3%) experienced 4-h SCs and 14 patients (40.0%) experienced 24-h SCs, while one patient in each group experienced SE. Overall, six (8.0%) and five (14.3%) patients required intravenous diazepam, midazolam, or phenobarbital, respectively, in the URW and RW group. No between-group differences were observed in terms of FBTCS, 4-h SCs, 24-h SCs, SE, or rescue medication use, and no falls or seizure-related injuries, such as fractures or epidural hematoma were reported. FBTCS and SC in the two groups were compared before and during LTM (Figure 2). ASM withdrawal may increase patient’s risk of SC, but this did not reach statistical significance in both groups. Additionally, not all patients with a history of FBTCS presented as such during LTM, with two patients experienced their first-ever FBTCS in the URW group and one patient in the RW group. Many patients in our study had no adverse events during the one-month follow-up after discharge.

## Discussion

Currently, no specific guidelines or expert consensus exist on ways to capture seizures effectively and safely in patients undergoing preoperative evaluation and provide an electrophysiological basis for the location of epileptic foci. Previous studies have reported that medication withdrawal is an effective plan, but is associated with an increased risk of FBTCS, SC, and SE. This retrospective study was designed to explore a potential safe and effective regimen for ASMs withdrawal, especially in developing countries, while considering the economic benefit ratio to provide a basis for the formulation of medication withdrawal guidelines.

Audits of EMUs revealed wide variations among units in the handling of ASM withdrawal and provision of rescue medication, with several units lacking standardized preventive measures (3, 5, 13). Previous studies have suggested that ASM reduction should be individualized to take into account both drug- and patient-related factors (14, 15), including habitual seizure rate, history of status epilepticus or seizure clusters, and risk of withdrawal seizures (benzodiazepines and barbiturates). And the type of medicine, medication half-life and potential for tolerance were also important in withdrawal decisions. However, there is little published literature on the subject, and a better evidence base is needed to inform ASM reduction decisions in the EMU. Several studies have reported ASM tapering regimens for LTM (4, 7, 16), with few cases comparing seizure rates and adverse effects of slower versus faster discontinuation (17–19). Most studies used observational designs, and changes in patient populations and medication regimens make it difficult to extrapolate the utility and safety of different discontinuation rates from these studies. Only a few studies have detected differences in safety measures between different taper protocols.

Previous research tended to study patients who underwent slow or intermediate tapering rates of ASMs during LTM (2, 4, 8, 9, 20). However, in recent years more studies have reported on the rapid withdrawal of ASMs and even medication withdrawal at home, which aims to ensure the optimal use of EMU capacity, especially in the era of the coronavirus disease (COVID-19) pandemic (16, 19, 21–23). Therefore, in this study all ASMs were discontinued on admission for LTM in 75 patients, and the medications in the RW group were reduced by 50% daily in 35 patients. All patients included in the study completed the LTM within 2–6 days, although 20 patients (26.7%) in the URW group and five patients (14.3%) in the RW group did not experience any seizures. The LTM duration for rapid taper and slow taper of medications was significantly different in prior studies, but did not reach significance in the present study (*p* = 0.14) (16, 19). The main reasons for the difference were the different taper protocols and limitation with regards to the LTM duration in our EMU. Foong et al. found that 5 monitoring days were sufficient to capture the necessary number of events (24). LTM duration in many EMUs for pre-surgical evaluation is 4 to 5 days, lasting even 1 to 2 weeks in some centers (4, 17, 25). However, this is not economically practical for most institutes. Reducing monitoring by 1 day results in a cost cut of approximately 1,000 China Yuan (USD 140) in our EMU. Furthermore, the time to onset of the first seizure was shorter than that reported in many previous studies and was statistically different between the two groups.

The safety of rapid ASM withdrawal during LTM, especially related to FBTCS, SC, and SE, should not be ignored. In our study, approximately 40% patients experienced FBTCS and 24-h SCs, and one patient in each group experienced SE. Among them, five–six patients required intravenous medications, such as intravenous diazepam, midazolam, or phenobarbital, in each group. The majority of patients who presented with FBTCS, SC, and/or SE following the rapid withdrawal of medication had a history of these respective conditions. However, the occurrence of FBTCS, SC, and SE in previous studies varied widely, which may be related to the different medication withdrawal protocols and patient characteristics. The percentage of patients with SC (41.3 and 40.0%) and SE (1.3 and 2.9%) in this study was similar to that reported by Rose et al. (48.5% in SC and 3.0% about SE), and lower than that reported in several previous studies (6, 7, 17, 26). In general, SC is common when ASMs are discontinued during LTM, but may not be significantly related to the rate of drug withdrawal. The percentage of patients with FBTCS (30.7 and 40.0%) in our study was similar to that reported by Guld et al. (38.3%) and less than that reported by Yen et al. study (57.3%) (4, 17). In previous studies, FBTCS was significantly associated with previous FBTCS episodes and sudden unexpected death in epilepsy (20, 27). We compared FBTCS and SC in the two groups before and during LTM. In the URW group, two patients experienced their first-ever FBTCS, while in the RW group, one patient experienced their first-ever FBTCS. However, the longitudinal comparison span is relatively large, and the reliability of the results needs to be further verified. When such an adverse event occurs, administration of rescue medication is important to stop the seizures.

However, our study had several limitations. First, no direct comparison was made with a longer duration of LTM and slower tapering of ASMs. In addition, the small sample size, especially in the RW group, limited the statistical power of the study. Furthermore, there is a lack of data on incidences of SC and SE before admission and after discharge from the hospital. Some of the patient demographic data, such as age at seizure onset and duration of epilepsy, were not comparable, which may be related to the severity of epilepsy. Previous studies have suggested that ASM reduction should be individualized to take into account both drug- and patient-related factors and the baseline data, such as monthly seizure frequency, history of FBTCS, SC, and SE, did not reach statistical significance between groups in this study. Though seizures were not captured in a higher percentage of ultra-rapid withdrawal patients and the possibility of such selection bias cannot be ruled out, we believed that the effect of this selection bias on our results was limited. The patients included in this study were from our EMU; therefore, the generalizability of our findings remains to be confirmed.

## Conclusion

In summary, the results of this study indicate that withdrawal of ASMs was an effective and relatively safe approach to provoke seizures in both URW and RW patients. We found no significant differences in seizure clusters and status epilepticus between the groups, and the requirement for intravenous rescue medications was low. Compared to the use of rapid ASM withdrawal protocol, the use of an ultra-rapid ASM withdrawal protocol allowed us to capture more patients’ initial seizures within the first day of monitoring.

## Data availability statement

The original contributions presented in the study are included in the article/supplementary material, further inquiries can be directed to the corresponding author.

## Ethics statement

The studies involving human participants were reviewed and approved by the Ethics Committee of West China Hospital of Sichuan University. The patients/participants provided their written informed consent to participate in this study.

## Author contributions

JL, DC, and LL were involved in data design and interpretation. YX and YZ were involved in data collection. JL and LL drafted the manuscript. All authors contributed to the article and approved the submitted version.
